# Self-rated health in multimorbid older general practice patients: a cross-sectional study in Germany

**DOI:** 10.1186/1471-2296-15-1

**Published:** 2014-01-03

**Authors:** Anna Nützel, Anne Dahlhaus, Angela Fuchs, Jochen Gensichen, Hans-Helmut König, Steffi Riedel-Heller, Wolfgang Maier, Ingmar Schäfer, Gerhard Schön, Siegfried Weyerer, Birgitt Wiese, Martin Scherer, Hendrik van den Bussche, Horst Bickel

**Affiliations:** 1 Department of Psychiatry and Psychotherapy, Technical University of Munich, Munich, Germany; 2 Institute of General Practice, University of Frankfurt/Main, Frankfurt, Germany; 3 Institute of General Practice, University of Düsseldorf, Düsseldorf, Germany; 4 Institute of General Practice, University of Jena, Jena, Germany; 5 Department of Medical Sociology and Health Economics, University Medical Medical Center Hamburg-Eppendorf, Hamburg, Germany; 6 Institute for Social Medicine, Occupational Health and Public Health, University of Leipzig, Leipzig, Germany; 7 Department of Psychiatry and Psychotherapy, University of Bonn, Bonn, Germany; 8 Institute of Primary Medical Care, University Medical Center Hamburg-Eppendorf, Hamburg, Germany; 9 Institute of Medical Biometry and Epidemiology, University Medical Center Hamburg-Eppendorf, Hamburg-Eppendorf, Germany; 10 Central Institute of Mental Health, Medical Faculty Mannheim/Heidelberg University, Mannheim, Germany; 11 Institute for Biometry, Hannover Medical School, Hannover, Germany

**Keywords:** Quality of life, Self-assessment, Chronic disease, Depression, Pain, Functionally- impaired elderly, General practice

## Abstract

**Background:**

With increasing life expectancy the number of people affected by multimorbidity rises. Knowledge of factors associated with health-related quality of life in multimorbid people is scarce. We aimed to identify the factors that are associated with self-rated health (SRH) in aged multimorbid primary care patients.

**Methods:**

Cross-sectional study with 3,189 multimorbid primary care patients aged from 65 to 85 years recruited in 158 general practices in 8 study centers in Germany. Information about morbidity, risk factors, resources, functional status and socio-economic data were collected in face-to-face interviews. Factors associated with SRH were identified by multivariable regression analyses.

**Results:**

Depression, somatization, pain, limitations of instrumental activities (iADL), age, distress and Body Mass Index (BMI) were inversely related with SRH. Higher levels of physical activity, income and self-efficacy expectation had a positive association with SRH. The only chronic diseases remaining in the final model were Parkinson’s disease and neuropathies. The final model accounted for 35% variance of SRH. Separate analyses for men and women detected some similarities; however, gender specific variation existed for several factors.

**Conclusion:**

In multimorbid patients symptoms and consequences of diseases such as pain and activity limitations, as well as depression, seem to be far stronger associated with SRH than the diseases themselves. High income and self-efficacy expectation are independently associated with better SRH and high BMI and age with low SRH.

**Trial registration:**

*MultiCare Cohort study registration:*ISRCTN89818205.

## Background

Multimorbidity is an issue of increasing importance to the health care system. The prevalence of multimorbidity rises from 10 percent in the 0 to 19 year-olds up to 78 percent in people aged 80 years and older [1]. As a consequence of an increasing life expectancy, the number of people affected by multimorbidity will probably steadily grow. However, little is known about the impact of multimorbidity on health-related quality of life of elderly primary care patients.

A persons’ own subjective rating of their health status was found to be an important predictor of morbidity and mortality, as well as a useful indicator of health-related quality of life [2,3].

Factors associated with health-related quality of life have been studied in the general population as well as in patient-populations suffering from different chronic diseases. Several studies indicated a positive association between higher education and income with better self-rated health (SRH) [4-6]. The relationship between age, gender and SRH, however, is less clear [5-7]. Modifiable lifestyle factors, such as obesity, smoking, risky alcohol consumption and low levels of physical activity were found to have a negative correlation with health-related quality of life [8-10]. Furthermore, several studies concurrently report a negative association between depressive symptoms and chronic pain and SRH [11,12], respectively. In recent years, research has particularly focused on the relationship between different chronic diseases and health-related quality of life. Neurological diseases, cancer and rheumatoid arthritis have been reported as conditions associated with low SRH among the elderly [13]. With increasing age the prevalence of chronic diseases, disability and limitations of activities of daily living rises, with limitations of daily activities being associated with lower levels of SRH [14].

Although a lot is known about factors correlated with SRH in the general population, knowledge of corresponding factors in aged multimorbid patients is scarce. Important questions remain: Is SRH in multimorbid patients more strongly affected by the presence of single disease states or by the sequelae of illnesses (i.e. pain, limitations of daily living)? What are demographic (i.e. age, education and income), lifestyle (i.e. BMI, smoking, alcohol consumption, level of physical activity) and psychological factors (i.e. depression, social support and self-efficacy expectation) associated with SRH in this patient group? Is SRH determined by the same factors in men and women or are there gender-specific differences? Therefore, the aim of this study was twofold: First, to identify the factors that are independently related to SRH in a multimorbid primary care sample of elderly people; and second, to identify possible gender-related differences in these factors.

## Methods

### Study design

Multimorbidity is usually defined as the presence of two or more illnesses at the same time. In the inspection of the diagnosis distribution it however became clear that two or more chronic illnesses were present in practically all our elderly patients. We thereupon defined multimorbidity as the presence of at least three chronic illnesses. In addition, in order to ensure a large number of patterns of multimorbidity, the very frequent illnesses with a prevalence of over 25% (e.g. hypertension, hyperlipidemia) were not considered for the inclusion in the sample. Nevertheless, these highly prevalent diagnoses are frequently combined with the relatively lower prevalent ones and are therefore still part of the sample. A detailed list of the 29 diseases used for inclusion of multimorbid patients can be found elsewhere [15]. This list was newly compiled at the beginning of the MultiCare-Study and represents the most frequent chronic conditions in the population based on prevalence data.

Data analyzed in this study came from the baseline investigation of the German MultiCare-Study [15], conducted from July 2008 to October 2009.

Patients were recruited in 8 study centers across Germany (Bonn, Düsseldorf, Frankfurt/Main, Hamburg, Jena, Leipzig, Mannheim and Munich). In each city about 20 general practitiosners (GPs) were recruited and asked to provide the study group with a list containing all of their patients between 65 and 85 years (date of birth 1.7.1923 to 30.6.1943), who had at least one consultation in the most recent quarter.

In each surgery, approximately 50 patients of those who suffered from at least three different chronic diseases out of a reference list of 29 chronic conditions [15] and did not meet the exclusion criteria (see below), were drawn at random. Multimorbidity was determined by chart review. These patients were contacted and asked to participate.

Exclusion criteria were:

● Residence in a nursing home

● Severe illness probably lethal within three months according to the GP

● Insufficient ability to speak and read German language

● Insufficient ability to consent (e.g. due to dementia)

● Insufficient ability to participate in interviews (e.g. due to blindness, deafness)

● Patients with no regular consultations and therefore poorly known to the GP

● Participation in other studies

### Data collection

Patients who met the inclusion criteria and were willing to participate were visited at home or in the GP practice and interviewed by a trained investigator. A set of standardized questionnaires was used to collect variables belonging to the area of socio-demography, lifestyle, psychological and illness-related factors. Table 1 provides an overview of the standardized instruments. The GPs measured height and weight at the patients’ next routine consultation in the surgery.

**Table 1:** Description of the instruments

**Abbreviation of the instrument**	**Function and interpretation**
AUDIT-C	Alcohol Use Disorders Identification Test [20]; 3 items with 5 possible response categories (0 to 4 points); *interpretation*: total score (max. 12 points): ≤ 7 points no suspicion of an alcohol related disorder, ≥ 8 points: suspicion of an alcohol related disorder
Barthel-Index	Measures performance in basic activities of daily living [26]; 10 items with 3 categories each (0, 5 and 10 points); *interpretation*: total score (max. 100 points); 0 to 30 points: largely dependent from others, 35 to 80 points: need of care, 85 to 95: punctual need of care, 100: independent from others
BMI	Body-Mass-Index (weight (kg)/height (m^2^)); *interpretation*: BMI < 18.5: underweight, 18.5 to < 25: normal weight, 25 to < 30: overweight and > 30: obesity
VAS of EQ-5D	Visual analogous scale of the EuroQoL-5D (EQ-5D) [16] measures subjective health related quality of life on a scale from “0” representing the worst to “100” representing the best possible health status; *interpretation*: higher scores represent higher rates of subjective health
F-SOZU K-14	Social support questionnaire [21]; 14 items; 5 point scale; mean of the sum of all items; *interpretation*: high scores indicate high social support
GCPS	Graded chronic pain scale [25]; 8 items, scale 0 to 10; 2 total scores: Characteristic pain intensity and Disability score; *interpretation*: higher scores represent higher pain intensity and higher disability caused by pain respectively
GDS	Geriatric Depression Scale [23]; 15 items; 0 vs. 1 point per item; max. 15 points; *interpretation*: 0 to 5 points: unsuspicious, ≥ 6 points: depressive episode likely
IADL	Instrumental Activities of Daily Living [27]; 8 items; 0 vs. 1 point per item; total score: men: item 1–2 and 6–8 (max. 5 points), women: item 1–8 (max. 8 points); *interpretation*: males: score < 5: with limitations, females: score < 8: with limitations
IPAQ-7	International Physical Activity Questionnaire [19]; 6 items buildup 3 scores: time spent on vigorous activity (weighting coefficient 8.0), on moderate activity (weighting coefficient 4.0) and on walking (weighting coefficient 3.3); “Total Metabolic Equivalent Task (MET)-minutes/week” calculated as follows: minutes x weighting coefficient; interpretation: low scores indicating low and high scores indicating high physical activity
SWE	Self-Efficacy Scale [22]; 10 items on a four-point scale (1 to 4 points); total score: sum of the 10 items divided by 10; *interpretation*: high scores indicating high self-efficacy expectation
4 DBL	Four-dimensional symptom questionnaire [24]; 50 items with 5 point scale; 4 sub scores reflecting the factors “somatization” (16 items), “anxiety” (12 items), “depression” (6 items) and “distress” (16 items); *interpretation*: somatization >10: moderate, >20 high; anxiety: >8: moderate, >12: high; depression: >2: moderate, >5: high; general distress: >10: moderate, >20: high

### Self-rated health

SRH was measured with the visual analogous scale (VAS) of the EuroQoL-5D (EQ-5D) [16]. With one single question (“In general, how would you rate your health status today?”) patients were asked to rate their subjective health status on a scale from “0” representing the worst to “100” representing the best possible health status.

### Socio-demographic variables

Socio-demographic variables included age, gender, marital status and living conditions. Educational level was categorized as low, intermediate or high according to the CASMIN (Comparative Analysis of Social Mobility in Industrial Nations) classification [17], a certificate-orientated classification scheme developed by an international research group. Equivalent income was calculated from the total household income by using the per capita demand weighting scale [18] of the Organization for Economic Cooperation and Development (OECD). It was calculated as household total net income per month divided by the equivalised household size, which gives 1.0 to the householder, 0.5 to other household members aged 15 or over and 0.3 to each child aged less than 15 years old.

### Lifestyle variables

Lifestyle variables included physical activity (IPAQ-7) [19], alcohol consumption (AUDIT-C) [20], smoking behavior and Body Mass Index (BMI). Smoking behavior was assessed by asking the patient the following question: “Are you a regular smoker, occasional smoker, former smoker or non-smoker?”

### Psychological characteristics

Psychological variables ascertained in the patient-interview included perceived social support (F-SOZU K-14) [21], patients’ self-efficacy expectation (SWE) [22], depression (GDS) [23] and symptoms belonging to the four dimensions somatization, depression, anxiety and distress (4 DBL) [24].

### Illness related factors

Patients were asked for the presence of chronic diseases. The interviewer read 32 chronic diseases (Table 2) to the patients who respectively responded with either “yes” or “no” to indicate whether they did or did not suffer from a particular chronic disease. Further, pain intensity and pain associated disability (GCPS) [25] were assessed. Additionally, grade of independence in performing basic (Barthel-Index) [26] and instrumental activities of daily living (iADL) [27] were illness-related factors that were rated by the interviewer.

**Table 2:** Prevalence of self-reported diagnoses by gender and for the whole sample

**Diagnosis group**	**Total (n** **=** **3189)**	**Men (n = 1298)**	**Women (n = 1891)**	***p value***^**3**^
Hypertension	2307 (72.3%)	939 (72.3%)	1368 (72.3%)	n.s.
Joint arthrosis	2115 (66.4%)	718 (55.4%)	1397 (73.9%)	< 0.001
Chronic low back pain	1975 (62.0%)	700 (54.1%)	1275 (67.5%)	< 0.001
Lipid metabolism disorders	1460 (45.9%)	617 (47.6%)	843 (44.7%)	n.s.
Chronic ischemic heart disease	963 (30.3%)	549 (42.3%)	414 (22.0%)	< 0.001
Severe vision reduction	1396 (43.9%)	515 (39.7%)	881 (46.8%)	< 0.001
Prostatic hyperplasia	511 (20.8%)	511 (39.6%)	--	--
Diabetes mellitus	992 (31.2%)	479 (37.0%)	513 (27.2%)	< 0.001
Cardiac arrhythmia	1044 (32.8%)	451 (34.8%)	595 (31.5%)	n.s.
Neuropathies	1114 (34.9%)	426 (32.8%)	688 (36.4%)	0.041
Dizziness	1109 (34.8%)	381 (29.4%)	728 (38.5%)	< 0.001
Lower limb varicosis	1148 (36.0%)	321 (24.8%)	827 (43.8%)	< 0.001
Asthma/COPD^1^	696 (21.8%)	280 (21.6%)	416 (22.0%)	n.s.
Purine/pyrimidine metabolism disorders/Gout	536 (16.9%)	269 (20.8%)	267 (14.2%)	< 0.001
Haemorrhoids	727 (22.8%)	246 (19.0%)	481 (25.5%)	< 0.001
Cerebral ischemia/Chronic stroke	444 (13.9%)	225 (17.4%)	219 (11.6%)	< 0.001
Cardiac insufficiency	548 (17.2%)	214 (16.5%)	334 (17.7%)	n.s.
Atherosclerosis/PAOD^2^	347 (10.9%)	193 (14.9%)	154 (8.2%)	< 0.001
Thyroid dysfunction	991 (31.1%)	192 (14.8%)	799 (42.3%)	< 0.001
Cancer	332 (10.4%)	171 (13.2%)	161 (8.8%)	< 0.001
Noninflammatory gynaecological problems	246 (9.1%)	--	246 (13.1%)	--
Renal insufficiency	307 (9.6%)	150 (11.6%)	157 (8.3%)	0.003
Cardiac valve disorders	314 (9.9%)	139 (10.7%)	175 (9.3%)	n.s.
Intestinal diverticulosis	435 (13.7%)	138 (10.6%)	297 (15.7%)	< 0.001
Psoriasis	213 (6.7%)	117 (9.0%)	96 (5.1%)	< 0.001
Rheumatoid arthritis/Chronic polyarthritis	410 (12.9%)	112 (8.7%)	298 (15.8%)	< 0.001
Osteoporosis	690 (21.7%)	97 (7.5%)	593 (31.4%)	< 0.001
Chronic cholecystitis/Gallstones	271 (8.5%)	78 (6.0%)	193 (10.2%)	< 0.001
Urinary tract stones	124 (3.9%)	63 (4.9%)	61 (3.2%)	0.025
Anemia	169 (5.3%)	54 (4.2%)	115 (6.1%)	0.019
Migraine/chronic headache	166 (5.2%)	37 (2.9%)	129 (6.8%)	< 0.001
Parkinson’s disease	67 (2.1%)	36 (2.8%)	31 (1.6%)	0.032

Note: ^1^: chronic obstructive pulmonary disease; ^2^: peripheral arterial occlusive disease;

^3^: χ2-test, df = 1, two-sided p, n.s. = not significant (p > 0.05).

### Missing values

Missing values were substituted by the hot-deck method. This technique identifies the most similar case in the sample (nearest neighbor distance) and uses this value for imputation [28]. If more than one case were possible for the imputation one case was selected by chance. Imputation was done with the R - 2.13.0 package StatMatch [29]. A detailed description of the substitution process can be found elsewhere [30]. For all variables the missing value rate was less than 2 percent except the variable total household income which had a missing value rate of 12.2 percent.

### Data analysis

The data were analyzed using the Statistical Package for the Social Sciences (SPSS, Version 19.0). Means (*M*) and standard deviations (*SD*) were calculated for continuous variables and frequencies, as well as percentages, for categorical variables. Group differences were tested for statistical significance either by χ^2^-test or t-test as appropriate. Linearity of the relationship between independent variables and SRH was controlled by visual inspection. Bivariate associations between risk factors and SRH were analyzed by Pearson’s correlation and differences by t-test.

Variables that showed a significance level of *p* ≤ 0.01 in the bivariate analyses were entered into multivariable linear regression analyses in a stepwise forward manner, with SRH as the dependent variable. Multivariable linear regression analyses were performed for the whole sample as well as for men and women separately.

### Ethical considerations

The study protocol was approved by the Ethics Committee of the Medical Association of Hamburg (Approval Nr. 2881) and by the Ethics Committees of the participating study centers. Written consent was obtained from every participant after being completely informed about the study.

## Results

A total of 50,786 patients in the database of the participating GPs fulfilled the age criterion and had at least one GP contact in the last quarter. Out of those 24,862 were randomly selected and checked for the presence of at least three chronic diseases and exclusion criteria. After exclusion of the patients without multimorbidity and those who met the exclusion criteria, 7,172 patients remained and were contacted. Out of those 3,317 agreed to participate (response rate 46.2%). In total, data of 3,189 patients were included in the final analyses. The difference of 128 cases between the 3,317 patients who agreed to participate and the 3,189 whose data it was possible to include in the statistical analysis is due to the fact that patients died before they could be interviewed or that exclusion criteria became obvious only after sample selection. A more detailed description of sampling and response rate, as well as a non-responder analysis can be found elsewhere [30].

### Characterization of the study population

Table 3 summarizes the mean values in demographic, lifestyle and psychological variables separately for men and women. Women represented 59.3% of the study participants. Mean EQ-VAS value of the whole sample was 62.5 (SD = 18.2); men had a significant higher mean SRH of 63.6 (*SD* 18.4) compared to 61.6 (*SD* 18.0) in women (*p* = 0.003, see Table 4). The prevalence of self-reported diseases is presented in Table 2. Hypertension (present in 72.3%), joint arthrosis (present in 55.4% of the men and 73.9% of the women) and chronic low back pain (present in 54.1% of the men and 67.5% of the women) were the most frequently reported diseases in the study population.

**Table 3:** Characteristics of the study population

	**Total (n = 3189)**	**Men (n = 1298)**	**Women (n = 1891)**	**P value**^**2**^	
Self-rated health (SRH) (Mean (SD))	62.4 (18.2)	63.6 (18.4)	61.6 (18.0)	0.003	
**Demographic variables**					
Age, in years (Mean (SD))	74.4 (5.2)	74.0 (5.1)	74.7 (5.3)	0.001	
Marital status (N (%))					
*Married*	1863 (58.4%)	1026 (79.0)	837 (44.3)		
*Single*	188 (5.9%)	56 (4.3)	132 (7.0)		
*Divorced*	256 (8.0%)	74 (5.7)	182 (9.6)		
*Widowed*	882 (27.7%)	142 (10.9)	740 (39.1)	< 0.001	(df = 3)
Living conditions (N (%))					
*One person household*	1128 (35.4%)	229 (17.6)	899 (47.5)		
*Living with partner/spouse*	1847 (57.9%)	1021 (78.7)	826 (43.7)		
*Living with others*^*1*^	214 (6.7%)	48 (3.7)	166 (8.8)	< 0.001	(df = 2)
Education (N (%))					
*Low*	1986 (62.3%)	753 (58.0)	1233 (65.2)		
*Intermediate*	856 (26.8%)	306 (23.6)	550 (29.1)		
*High*	347 (10.9%)	239 (18.4)	108 (5.7)	< 0.001	(df = 2)
Monthly income (in Euro) (Mean (SD))	1412.2 (705.9)	1517.0 (833.0)	1340.3 (593.0)	< 0.001	
**Lifestyle variables**					
Smoking behavior (N (%))					
*Current smoker*	292 (9.2%)	153 (11.8)	139 (7.3)		
*Former smoker*	1361 (42.7%)	819 (63.1)	542 (28.7)		
*Non smoker*	1532 (48.0%)	324 (25.0)	1208 (63.9)	< 0.001	(df = 2)
Body mass index (Mean (SD))	28.2 (4.9)	28.1 (4.0)	28.3 (5.4)	n.s.	
Alcohol habits score (Mean (SD))	2.2 (1.9)	3.0 (2.2)	1.6 (1.5)	< 0.001	
Physical activity (in 1000 MET minutes/week) (Mean (SD))	2.2 (2.5)	2.6 (2.8)	1.9 (2.2)	< 0.001	
**Psychological variables**					
Self-efficacy (SWE) (Mean (SD))	3.3 (0.6)	3.4 (0.5)	3.2 (0.6)	< 0.001	
Social support (F-SOZU) (Mean (SD))	4.1 (0.7)	4.1 (0.7)	4.1 (0.7)	n.s.	
Depression (GDS) (Mean (SD))	2.6 (2.6)	2.3 (2.5)	2.8 (2.7)	< 0.001	
Somatization (4DBL) (Mean (SD))	7.0 (5.1)	5.7 (4.7)	7.8 (5.2)	< 0.001	
Anxiety (4DBL) (Mean (SD))	1.0 (2.2)	0.6 (1.7)	1.3 (2.4)	< 0.001	
Depression (4DBL) (Mean (SD))	0.8 (2.0)	0.7 (1.8)	1.0 (2.1)	< 0.001	
Distress (4DBL) (Mean (SD))	5.9 (5.3)	4.7 (4.7)	6.8 (5.5)	< 0.001	
**Disease-related variables**					
Characteristic pain intensity (GCPS) (Mean (SD))	34.5 (25.5)	28.1 (24.7)	39.0 (25.2)	< 0.001	
Disability score (GCPS) (Mean (SD))	26.0 (29.2)	20.2 (27.3)	30.0 (29.2)	< 0.001	

^1^: Living with others: including living together with other family members or other persons and living in assisted living or in retirement home; ^2^ χ^2^-test or t-test as appropriate, two-sided p, n.s. = not significant (p > 0.05), df: degrees of freedom.

**Table 4:** Self-rated health of the whole sample in relation to demographic variables and self-reported diseases

**Variables**	**Mean (N)**	**SD**	**Mean (N)**	**SD**	***p *** **value**	**Effect size**
**Self-reported diseases**	** Present**	** Absent**				
Hypertension	61.7 (2307)	18.0	64.3 (882)	18.5	<0.001	0.14
Joint arthrosis	60.0 (2115)	17.9	67.2 (1072)	17.8	<0.001	0.40
Chronic low back pain	59.1 (1975)	18.2	68.0 (1208)	16.9	<0.001	0.49
Chronic ischemic heart disease	59.4 (963)	18.8	63.7 (2220)	17.8	<0.001	0.24
Severe vision reduction	60.8 (1396)	18.3	63.7 (1786)	18.0	<0.001	0.12
Diabetes mellitus	61.0 (992)	18.0	63.1 (2188)	18.3	0.004	0.12
Cardiac arrhythmia	59.0 (1046)	18.2	64.1 (2139)	18.0	<0.001	0.28
Neuropathies	57.1 (1114)	18.2	65.2 (2075)	17.6	<0.001	0.45
Dizziness	57.1 (1109)	17.9	65.3 (2078)	17.7	<0.001	0.45
Lower limb varicosis	61.1 (1148)	18.4	63.2 (2037)	18.0	0.002	0.12
Asthma/COPD^1^	58.0 (696)	18.5	63.7 (2491)	17.9	<0.001	0.31
Purine/pyrimidine metabolism disorders/Gout	57.7 (536)	18.1	63.4 (2643)	18.1	<0.001	0.31
Hemorrhoids	59.9 (727)	18.4	63.2 (2459)	18.1	<0.001	0.18
Cerebral ischemia/Chronic stroke	59.3 (444)	18.4	62.9 (2742)	18.1	<0.001	0.20
Cardiac insufficiency	55.7 (548)	18.4	63.8 (2632)	17.8	<0.001	0.45
Atherosclerosis/PAOD^2^	54.6 (347)	19.9	63.4 (2835)	17.8	<0.001	0.48
Renal insufficiency	55.1 (307)	19.1	63.2 (2875)	17.9	<0.001	0.45
Intestinal diverticulosis	60.8 (435)	17.7	62.7 (2751)	18.3	0.046	0.10
Rheumatoid arthritis	55.0 (410)	18.8	63.5 (2768)	17.8	<0.001	0.47
Osteoporosis	57.4 (690)	18.7	63.8 (2496)	17.8	<0.001	0.35
Chronic cholecystitis/Gallstones	59.1 (271)	18.4	62.7 (2917)	18.2	0.002	0.20
Anemia	55.2 (169)	18.1	62.8 (3017)	18.1	<0.001	0.42
Migraine	58.8 (166)	18.6	62.6 (3022)	18.2	0.008	0.21
Parkinson’s disease	49.5 (67)	18.4	62.7 (3121)	18.1	<0.001	0.73
Lipid metabolism disorders	61.8 (1460)	17.9	62.9 (1721)	18.4	0.10	0.06
Thyroid dysfunction	61.7 (991)	18.2	62.7 (2196)	18.2	0.15	0.05
Prostate hyperplasia	63.3 (510)	17.7	63.8 (779)	18.8	0.63	0.03
Non-inflammatory gynecological problems	60.8 (245)	19.7	61.7 (1631)	17.8	0.45	0.05
Cardiac valve disorders	61.4 (314)	18.4	62.5 (2872)	18.2	0.31	0.06
Kidney stones	60.7 (124)	18.5	62.5 (3063)	18.2	0.29	0.10
Psoriasis	61.4 (213)	19.2	62.5 (2976)	18.1	0.39	0.06
Malignant tumors	60.9 (332)	18.0	62.6 (2850)	18.2	0.11	0.09
**Sex**	** Males**	** Females**				
	63.6 (1298)	18.4	61.6 (1891)	18.0	0.003	0.11
**Marital status**	** Married**	** Non married**				
	63.3 (1863)	18.2	61.2 (1326)	18.2	0.001	0.12
**Household type**	** With others**		** Living alone**			
	63.0 (2061)	18.2	61.3 (1128)	18.2	0.011	0.09
**Education**	** High**	** Low/Intermediate**				
	67.1 (347)	18.7	61.8 (2842)	18.1	<0.001	0.29
**Smoking behavior**	** Smoker**	** Non-smoker**				
	61.5 (237)	18.4	62.5 (2948)	18.2	0.42	0.05

Note: By t-test differences in SRH between different groups were tested for significance; confidence interval: 99%; only significant results are shown in the table.

Marital status: not married includes: single, divorced, widowed; household type: living with others includes: with partner, family members and in an institution; ^1^: chronic obstructive pulmonary disease; ^2^: peripheral arterial occlusive disease.

### Bivariate analyses

The bivariate analyses suggested that all continuous variables showed weak but highly significant (*p* < 0.001) correlations with the dependent variable SRH (Table 5). Similarly, for all demographic and lifestyle variables and for most individual chronic diseases significant differences in SRH values were found. Table 4 presents differences between groups. Age and female gender was associated with lower SRH values and income, education, being married and living together with others are socio-demographic factors that were positively associated with SRH. A negative association was found between depression, anxiety, somatization and mental distress and SRH. Self-efficacy expectation and social support were psychological factors positively associated with SRH. Among the variables which showed no significant differences in mean SRH values were smoking and the presence or absence of lipid metabolism disorders, thyroid dysfunction, prostate hyperplasia, non-inflammatory gynecological problems, cardiac valve disorders, kidney stones, psoriasis and malignant tumors.

**Table 5:** Correlation (Pearson correlation coefficient (r)) of patient characteristics with self-rated health

**Characteristics**	**Men (n = 1298)**	**Women (n = 1891)**	**Total (n = 3189)**
**Socioeconomic variables**			
Age	−0.08 **	−0.13 ***	−0.11 ***
Income	0.12 ***	0.13 ***	0.13 ***
**Lifestyle variables**			
Body mass index (BMI)	−0.13 ***	−0.16 ***	−0.15 ***
Alcohol habit score (AUDIT-C)	0.10 **	0.13 ***	0.12 ***
Physical activity (IPAQ-7)	0.26 ***	0.22 ***	0.24 ***
**Psychological variables**			
Self-efficacy (SWE)	0.24 ***	0.20 ***	0.22 ***
Social support (F-SOZU)	0.15 ***	0.20 ***	0.18 ***
Depression (GDS)	−0.42 ***	−0.40 ***	−0.41 ***
Somatization (4DBL)	−0.42 ***	−0.43 ***	−0.43 ***
Anxiety (4DBL)	−0.26 ***	−0.22 ***	−0.23 ***
Depression (4DBL)	−0.27 ***	−0.30 ***	−0.29 ***
Distress (4DBL)	−0.36 ***	−0.37 ***	−0.37 ***
**Disease-related variables**			
Basic activities of daily living (Barthel)	0.29 ***	0.23 ***	0.25 ***
Instrumental activities (IADL)	0.29 ***	0.27 ***	-- ^1^
Characteristic pain intensity (GCPS)	−0.39 ***	−0.42 ***	−0.41 ***
Pain Disability score (GCPS)	−0.43 ***	−0.47 ***	−0.46 ***

Note: *** *P* values are <0.001; ** *P* values are <0.01; ^1^ correlation for the whole sample not shown because of different numbers of items for men and women, respectively.

### Multiple regression analyses

Table 6 shows the factors remaining in the final regression model when the whole sample was analyzed. These factors explained 35% of the variance in SRH. The intensity of chronic pain, pain associated disability, depressive symptoms, somatization, high BMI levels (all *p* < 0.001), age (*p* = 0.001), psychological distress (*p* = 0.01), a self-reported diagnosis of Parkinson’s disease (*p* = 0.003) and neuropathies (*p* = 0.008) had a significant negative effect on SRH. Significantly positive relationships were found between SRH and physical activity, independency in instrumental activities, higher monthly income (all *p* < 0.001) and self-efficacy expectation (*p* = 0.002).

**Table 6:** Correlates of self-rated health in the whole sample: results of a stepwise linear multiple regression model

	**Regression coefficient B (95% CI)**	**Standardized coefficient (beta)**	***p value***
Disability score (GCPS)	−0.11 (−0.15/-0.08)	−0.18	<0.001
Depression (GDS)	−0.96 (−1.31/-0.60)	−0.14	<0.001
Somatization (4DBL)	−0.61 (−0.79/-0.43)	−0.17	<0.001
Physical activity (IPAQ-7) (in 1000 MET minutes/week)	1.00 (0.38/0.97)	0.09	<0.001
Instrumental activities (IADL)	1.11 (0.70/1.51)	0.11	<0.001
Characteristic pain intensity (GCPS)	−0.07 (−0.11/-0.03)	−0.09	<0.001
Monthly income (in 1000 Euro)	2.00 (0.56/2.51)	0.06	<0.001
BMI	−0.24 (−0.38/-0.09)	−0.06	<0.001
Age	−0.18 (−0.32/-0.05)	−0.05	0.001
Self-efficacy (SWE)	1.60 (0.27/2.94)	0.05	0.002
Parkinson’s disease	−5.72 (−10.63/-0.80)	−0.04	0.003
Neuropathies	−1.58 (−3.12/-0.04)	−0.04	0.008
Distress (4DBL)	−0.19 (−0.37/0.00)	−0.05	0.010

Note: R^2=^ .35; variables are listed in order of inclusion in the model.

Factors associated with SRH when multiple regression analyses were conducted for men and women separately are presented in Table 7. Separate analyses were able to explain equal amounts of variance in SRH in both genders. In men, seven variables explained 34% of the variance of SRH, whereas, in women eleven variables were found to explain 35% of the variance of SRH. In both genders depression, somatization and pain associated disability had a negative effect on SRH (all *p* < 0.001). Physical activity (*p* < 0.001) had a positive effect on SRH. Low or intermediate education (*p* < 0.001) and a self-reported diagnosis of cardiac arrhythmia (*p* = 0.004) were associated with reduced SRH in men. In women pain intensity, high BMI, distress (all *p* < 0.001), age (*p* = 0.001) and chronic low back pain (*p* = 0.004) were significantly negatively associated with SRH. SRH was better among women with higher monthly income (*p* = 0.009).

**Table 7:** Factors associated with SRH in males and females respectively

	**Men**			**Women**		
	**Regression coefficient B (95% CI)**	**Standar-dized coefficient (beta)**	***p value***	**Regression coefficient B (95% CI)**	**Standar-dized coefficient (beta)**	***p value***
Somatization (4DBL)	−0.79 (−1.00/-0.59)	−0.20	<0.001	−0.54 (−0.72/-0.37)	−0.16	<0.001
Disability score (GCPS)	−0.14 (−0.18/-0.11)	−0.21	<0.001	−0.10 (−0.14/-0.07)	−0.17	<0.001
Depression (GDS)	−1.51 (−1.90/-1.12)	−0.20	<0.001	−0.91 (−1.24/-0.58)	−0.13	<0.001
Physical activity (in 1000 MET minutes/week) (IPAQ-7)	1.00 (0.53/1.14)	0.13	<0.001	1.00 (0.28/0.94)	0.07	<0.001
Activities of daily living (Barthel)	0.30 (0.16/0.44)	0.10	<0.001	n.s.		
Low or intermediate education	−4.43 (−6.57/-2.29)	−0.09	<0.001	n.s.		
Cardiac arrhythmia	−2.57 (−4.34/-0.80)	−0.07	0.004	n.s.		
Characteristic pain intensity (GCPS)	n.s.			−0.07 (−0.11/-0.03)	−0.10	<0.001
Instrumental activities (IADL)	n.s.			1.09 (0.42/1.75)	0.07	0.001
BMI	n.s.			−0.24 (−0.37/-0.11)	−0.07	<0.001
Distress (4DBL)	n.s.			−0.30 (−0.47/-0.14)	−0.09	<0.001
Age	n.s.			−0.24 (−0.38/-0.11)	−0.07	0.001
Chronic low back pain	n.s.			−2.36(−3.97/-0.74)	−0.06	0.004
Monthly income (in 1000 Euro)	n.s.			2.00 (0.39/0.27)	−0.05	.009

Note: all variables that have shown significant results in the bivariate analyses were put in the model; males: R^2^ = 0.34;

females: R^2^ = 0.35.

In both genders a negative association between SRH and restrictions in activities of daily living was found. Whereas in men lower SRH scores were associated with restrictions in basic activities of daily living (*p* < 0.001), in women restrictions in instrumental activities (*p* < 0.001) resulted in significantly lower SRH values.

## Discussion

The present study aimed to identify socio-demographic, lifestyle, psychological and disease-related factors associated with SRH in a large sample of elderly multimorbid general practice patients. To the best of our knowledge there are no studies that investigated SRH in multimorbid samples of comparable size and age.

In our sample the mean EQ-VAS score was slightly below the general population’s mean score [6,31-34], but above values found in samples of chronically ill patients [31,34,35].

Whereas in the bivariate analyses nearly all variables showed significant correlations with SRH, the final regression model for the whole sample included 13 of the initially more than 50 variables. Lifestyle variables like current smoking and alcohol consumption [36,37] that were often found to influence SRH were not independently associated with SRH in our sample. But BMI and physical activity were lifestyle factors independently associated with SRH in our study. We found that increasing BMI reduced and physical activity increased SRH, respectively. The finding of high BMI as a correlate of low SRH was confirmed by other studies [38,39], but not by all [40]. Several studies support the strong relationship between high levels of physical activity and better SRH [36,41-43].

In our multimorbid sample the association between socioeconomic factors and SRH seems to be weaker than in other samples [36]. Age and income were the only socioeconomic variables independently correlated with SRH in the whole sample.Among the psychological factors depression, somatization and distress significantly reduced SRH whereas self-efficacy expectation increased SRH. It might be that in a sample of multimorbid elderly patients somatization probably reflects true physical symptoms representing diseases rather than unspecific somatic complaints. The association between mental distress [44-46] and depression [42,47-49] and SRH was shown in many studies. We suspect that psychological factors exist that mediate the subjective rating of health in patients suffering from multiple chronic diseases. Those with high expectations of self-efficacy and low levels of stress and depression probably cope better with multimorbidity.

Not surprisingly, among the disease-related factors single diseases were less influential for SRH in our sample than sequelae of diseases like pain intensity, disability caused by pain and restrictions in instrumental activities. It appears as if general factors like pain, disability, depression, somatic complaints, restrictions in physical activity and independent living, which accumulate in multimorbid patients due to the presence of multiple chronic diseases, affect SRH more than single self-reported diseases. Based on the fact that every patient in our sample having at least three diagnoses, it is not surprising that most diseases do not contribute independently to the explained variance of SRH. It might be, that in a general population sample with a lower prevalence of diagnoses the result would be different.

Gender-specific analyses indicated that there are no differences in the relationship between somatization, depression, pain associated disability, and low physical activity and SRH in men and women. It seems that in both genders consequences of and complaints due to multimorbidity explain most of the variance of SRH. Besides these most important factors, we found different variables to be associated with SRH specifically in men and women. As found in a previous study [50], education was associated with SRH only in men. A possible explanation might be that in most families the total household income is more dependent on men’s than on women’s educational level. BMI values revealed a negative association with SRH exclusively in women, in line with a study from the US which showed a stronger association between high BMI values and low SRH scores in aged women compared to aged men [51]. Chronic low back pain and characteristic pain intensity were negatively related to SRH exclusively in women. Therefore, it appears that in women SRH is more affected by pain than in men.

### Implications

Most importantly, we found SRH to be predominantly associated with modifiable factors. This suggests that SRH could be improved through specific interventions at the level of primary care. Main focus should be on modifiable aspects and consequences of multimorbidity: appropriate interventions of pain treatment and reduction; thorough exploration of somatic symptoms, since they could reflect sequelae of multimorbidity as well as potential side and interaction effects of polypharmacy. In order to improve SRH, physical complaints should be relieved, be it by improving patients compliance to or by adjusting the medication. Besides the reduction of pain and somatic complaints, depression provides another important starting point for improvement of SRH. The high burden of physical complaints and symptoms could make it difficult to reliably detect and diagnose depression in elderly multimorbid patients. Screening for and when indicated, treatment of depression should be standard in multimorbid patients. In addition, patients’ health-related behavior should be the target of interventions in order to improve SRH. Possible interventions are: participation on special training programs for elderly that focus on weight reduction and simultaneously increase physical activity; rehabilitation interventions for those patients who have restrictions in functional abilities.

### Strengths and weaknesses

A major strength of our study was the large number of multimorbid general practice patients assessed that were spread over eight study centers distributed throughout Germany. In contrast to other studies of comparable size, which are generally based on postal or telephone surveys, our study data was collected by face-to-face interviews. In our study a larger set of variables was collected and tested for associations than in comparable studies. To enhance accuracy of the diagnoses used for inclusion of multimorbid patients, GPs’ diagnoses were used for selection of participants. Nevertheless, GPs’ diagnoses are also not entirely valid [52]. Despite the fact that participants were of advanced age and suffering from multimorbidity we obtained a satisfactory response rate.

We decided to measure the health related life quality by means of a global visual analog scale. To this it could be objected that such a simple scale were not capable of representing all facets of the complex construct: life quality. According to Idler and Benyamini [2], however, it was possible to show that global self-ratings of health reflect “the respondents’ views of global health in a way that nothing else can” (p. 34). We used an analog scale in order to allow the patients to include their own dimensions into their concept of health-related quality-of-life.

However, the present study also had some limitations. Most of the information was obtained through self-report, which may reduce the validity of the information. For example, questions about alcohol consumption, smoking behavior and physical activity may have been answered in a socially acceptable manner. Conversely, it can be seen as an advantage of our study that information regarding BMI was directly measured by the physician and the ability to perform instrumental and basic activities was rated by the interviewer based on the patients’ narration. Although electronic health records are more reliable in detecting multimorbidity in younger patients, the prevalence of multimorbidity in elderly patients appears to be the same for electronic health records as for self-reports [53]. Patients living in a nursing home and those suffering from dementia or severe illness of terminal stage were excluded from the sample. Therefore, it is possible that certain aspects of multimorbidity are not represented in our sample. Due to the cross-sectional character of our data, the direction of the relationship between SRH and independent factors remains unclear.

## Conclusions

SRH is a pivotal indicator of quality of life. The identification of factors influencing health-related quality of life in elderly multimorbid patients gains in importance in our aging society.

We found the strongest correlation between SRH and disease sequelae, but only few diseases and only those with a high burden of symptoms or limitations were independently associated with low SRH. In women SRH seemed to be more strongly associated with pain, whereas, in men threats of limitations of activity seemed to play a larger role.

In conclusion, perception of health and health-related quality of life at an older age might be improved by treatment of disease sequelae such as pain and discomfort. To enhance quality of life in the elderly, particular attention might be paid to the diagnosis and treatment of depression.

## Abbreviations

AUDIT-C: Alcohol use disorders identification test; BMI: Body mass index; CASMIN: Comparative analysis of social mobility in industrial Nations; COPD: Chronic obstructive pulmonary disease; EQ-5D: EuroQoL-5D; F-SOZU K-14: Fragebogen zur sozialen Unterstützung; GCPS: Graded chronic pain scale; GDS: Geriatric depression scale; GPs: General practitioners; iADL: Instrumental activities of daily living; IPAQ-7: International physical activities questionnaire; M: Mean; OECD: Organization for economic cooperation; PAOD: Peripheral arterial occlusive disease; SD: Standard deviation; SPSS: Statistical package for the social sciences; SRH: Self-rated health; SWE: Self-efficacy expectation; VAS: Visual analogous scale; 4DBL: Four dimensional symptom questionnaire.

## Competing interests

The authors declare that they have no competing interests.

## Authors’ contributions

AN and HB performed the statistical analyses and interpretation of the data, and drafted the manuscript. IS, BW, MS and HvB conceived and designed the study. GS and BW prepared the data for analysis. AD, AF, JG, HHK, SRH, WM and SW participated in study design and implementation. All authors read and approved the final manuscript.

## Pre-publication history

The pre-publication history for this paper can be accessed here:

http://www.biomedcentral.com/1471-2296/15/1/prepub
